# Use of Simulator-Based Teaching to Improve Medical Students’ Knowledge and Competencies: Randomized Controlled Trial

**DOI:** 10.2196/jmir.9634

**Published:** 2018-09-24

**Authors:** Quentin Fischer, Yannis Sbissa, Pascal Nhan, Julien Adjedj, Fabien Picard, Alexandre Mignon, Olivier Varenne

**Affiliations:** 1 Department of Cardiology Cochin Hospital (Assistance Publique–Hôpitaux de Paris) Paris Descartes University Paris France; 2 Faculty of Medicine Paris Descartes University Paris France; 3 Surgical Intensive Care Unit Cochin Hospital (Assistance Publique–Hôpitaux de Paris) Paris Descartes University Paris France; 4 iLumens Department of Medical Simulation Paris Descartes University Paris France

**Keywords:** education, coronary angiography, high fidelity simulation training

## Abstract

**Background:**

Simulator-based teaching for coronary angiography (CA) is an attractive educational tool for medical students to improve their knowledge and skills. Its pedagogical impact has not been fully evaluated yet.

**Objective:**

The aim of this study was to compare traditional face-to-face teaching with a simulator-based teaching for the acquisition of coronary anatomy knowledge and CAs interpretation.

**Methods:**

A total of 118 medical school students in their fourth to sixth year were prospectively randomized in 2 groups: (1) a control teaching group (n=59, CONT group) and (2) a simulator group (using the Mentice VIST-Lab CA simulator; n=59, SIM group). The CONT group received a PowerPoint-based course, whereas the SIM group received a simulator-based course including the same information. After the course, all students were evaluated by 40 multiple choice questions (maximum of 100 points), including questions on coronary anatomy (part 1), angiographic projections (part 2), and real CAs interpretation (part 3). Satisfaction of the students was also evaluated by a simple questionnaire.

**Results:**

Student characteristics were identical in both the groups: 62/118 (52.5%) were female and age was 22.6 (SD 1.4) years. Moreover, 35.6% (42/118) were in their fourth year, 35.6% (42/118) were in the fifth year, and 28.8% (34/118) in the sixth year. During the evaluation, SIM students had higher global scores compared with CONT students, irrespective of their year of medical school (59.5 [SD 10.8] points vs 43.7 [SD 11.3] points, *P*<.001). The same observations were noted for each part of the test (36.9 [SD 6.6] points vs 29.6 [SD 6.9] points, *P*<.001; 5.9 [SD 3.0] points vs 3.1 [SD 2.8] points, *P*<.001; and 16.8 [SD 6.9] points vs 10.9 [SD 6.5] points, *P*<.001; for parts 1, 2, and 3, respectively). Student satisfaction was higher in the SIM group compared with the CONT group (98% vs 75%, *P*<.001).

**Conclusions:**

This study suggests that simulator-based teaching could potentially improve students’ knowledge of coronary anatomy, angiography projections, and interpretation of real clinical cases, suggesting better clinical skills. These results should encourage further evaluation of simulator-based teaching in other medical specialties and how they can translate into clinical practice.

## Introduction

Simulator-based training is booming in surgery, medical, and technical specialties, especially in cardiology.

The advantages of this technology have been recognized by numerous teaching consortiums, most notably, the Accreditation Council of Graduate Medical Education, which recommends simulation training for numerous specialties, as well as the Society for Cardiovascular Angiography and Intervention [1].

Two main types of simulation need to be differentiated according to their degree of immersion (high-fidelity and low-fidelity). Fidelity refers to the degree to which a model reproduces the state of a real-world object, feature, or condition. Improved technology leads to the development of an increasing number of high-fidelity simulators that more accurately mimic the real environment [2].

Some studies have shown improvements in residents’ performances after catheter-based interventions using high-fidelity simulation, with better scores for residents who were provided simulations than for those who were not [3]. Unlike traditional teaching, skills obtained through virtual reality simulation training, such as the translation of a 2-dimensional video image into a 3-dimensional (3D) working area or tactile feedback, could be transferred into clinical practice [4].

However, these high-fidelity simulators are more expensive than their low-fidelity counterparts, not only in terms of the acquisition cost but also by adding the related costs associated with the personnel and resources needed to use them, and their real pedagogical impact has not been rigorously evaluated.

The aim of this study was to teach medical students coronary arteries anatomy and angiography interpretation into clinical practice and to evaluate simulator-based teaching by a head-to-head comparison between a traditional teaching approach and a high-fidelity coronary angiography (CA) simulator.

## Methods

### Population

All participants were medical students at Paris Descartes University in their fourth, fifth, or sixth year, and none of them had experience in interventional cardiology. They voluntarily agreed to take part in the study, which was conducted at the Institute for Therapy Advancement under the auspices of the foundation, iLumens.

The institute is a private organization that provides simulation equipment and training staff to the Paris Descartes University and iLumens foundation, including a CA simulator specifically designed for academic training.

### Interventional Cardiology Simulator

**Figure 1:**
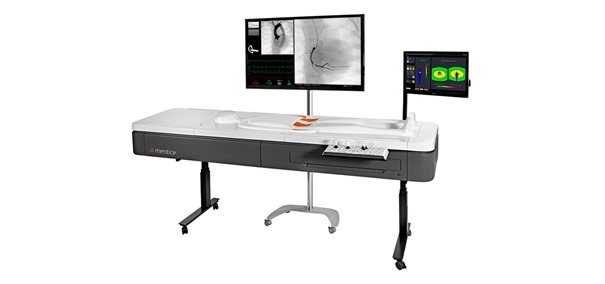
Mentice VIST-Lab simulator used in the study.

The Mentice VIST-Lab CA simulator (Mentice, Göteborg, Sweden) is a high-fidelity interface that includes a mannequin, 2 monitors, and joysticks for table and sensor control, mimicking the latest generation catheterizations laboratories. The simulator features buttons for zooming in and out, pedals for fluoroscopy, and cine loop control (see Figure 1).

The interventional tools, x-ray, and cine loops are all simulated to produce a highly realistic environment [5]. Injecting air using a syringe creates a virtual contrast injection. Users are able to switch between the angiographic view and a 3D view to better identify the take-off and location of the coronary arteries. The simulator includes several coronary and aortic anatomies and coronary stenosis. For the purpose of this study, each student worked on a single case.

### Sequence of the Session

All students were prospectively randomized by manual draw into 2 groups, regardless of their year of study: a control teaching group (n=59, CONT group) and a simulator group (n=59, SIM group). No pretesting was performed in our population, as we assumed that the students had the same level of knowledge in each group, because of the randomization. The CONT group received a PowerPoint-based course by an academic senior cardiologist, consisting of 15 slides encompassing the predefined learning objectives (coronary anatomy, angiography projections, and interpretation of real cases). The SIM group received a simulator-based course by the same cardiologist, which included the same pedagogical content. In addition to the theoretical course, all students in the SIM group were allowed to individually manipulate the simulator for 15 min to learn coronary anatomy in the real 3D environment. Several sessions were run because each one, both traditional and simulation, was conducted with small numbers of 8 to 10 students, to allow access to the simulator to all students and to allow time for questions and answers. The instructions, both visual and oral, delivered during the 2 types of teaching sessions were identical, as was the duration of the sessions (30 min).

### Evaluation

After the courses, the students were evaluated by a series of 40 multiple choice questions (MCQs) for a maximum of 100 points. For each MCQ, students had 5 choices. The MCQs were separated into 3 parts.

The first part was designed to evaluate coronary artery anatomy on still images and consisted of 25 MCQs. There was only 1 correct answer per MCQ in this part, and each correct choice scored 2 points, totaling 50 possible points for this part. This part was designed to promote traditional teaching because the same still images were displayed during the PowerPoint course.

The second part evaluated spatial representation on still pictures. The students had to distinguish between the right anterior oblique and left anterior oblique views and between caudal and cranial views. This part consisted of 5 MCQs, and each correct choice gave 2 points, giving a possible total of 10 points. There were 2 correct answers for each MCQ with binary notation.

**Figure 2:**
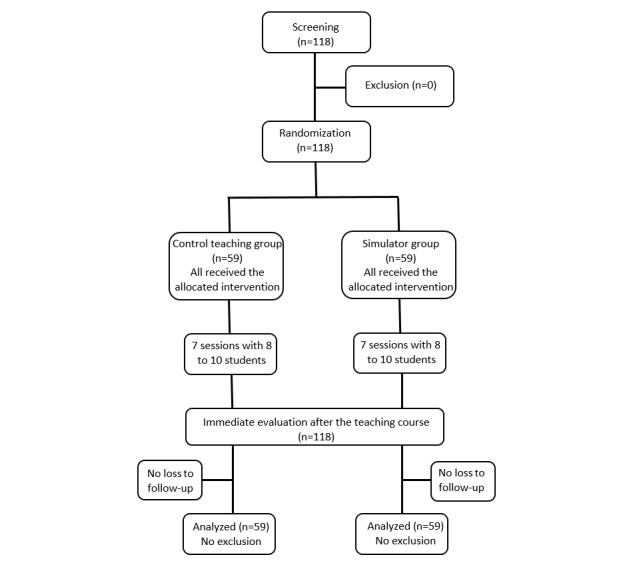
Flowchart of study population.

Although students in both the groups had received relevant training to be able to do this part, the 3D manipulation environment was potentially favoring the simulator group.

The third part evaluated interpretation of clinical cases–based angiographic films. There were 10 clinical scenario and 10 angiographies, each with 6 sequences to analyze. Each angiography corresponded to an MCQ and was played 3 times, twice at normal speed and once at a slow speed to help interpretation. The students were asked to choose between 5 possible answers reflecting clinical decisions. There was one or more correct answers for each MCQ with a binary notation. Each correct answer gave 4 points, giving a possible total of 40 points for this part.

The MCQs were accessible by logging onto a specific website, and the pictures and movies were presented by an external evaluator to prevent evaluation bias. The duration of the evaluation was the same for both the groups (1 hour).

Finally, student satisfaction was evaluated at the end of each session, with a binary notation (yes or no). Results of the evaluation were blindly analyzed, regardless of the inclusion group. Due to the design of the study, there was no crossover and no loss to follow-up (see Figure 2).

### Statistical Analysis

Continuous data are presented as mean (SD) and compared with the use of a Student *t* test. Categorical data are expressed as percentages and compared using a chi-square test. No sample size calculation was performed, with the inclusion of all volunteering Paris Descartes University medical students between the fourth and the sixth year.

All analyses were performed using SPSS version 22.0 (SPSS Inc, Chicago, IL, USA). A significance level of .05 was used to test for statistical differences.

## Results

A total of 118 medical students were included in the study. Of these, 35.6% (42/118) were in their fourth year, 35.6% (42/118) in their fifth year, and 28.8% (34/118) in their sixth year. Baseline characteristics of the students were similar in both the groups at inclusion. A total of 52.5% (62/118) were female, and the mean age was 22.6 (SD 1.4) years. There were 21 fourth-year students both in the CONT and SIM groups, 21 fifth-year students in the CONT and the SIM groups, and 17 sixth-year students in both the groups (see Table 1).

The global score was not significantly different in the 3 groups based on the year of study: 50.3 (SD 11.4) points in fourth year, 51.3 (SD 10.9) points in fifth year, and 53.5 (SD 10.5) points in sixth year (*P*=.52).

**Figure 3:**
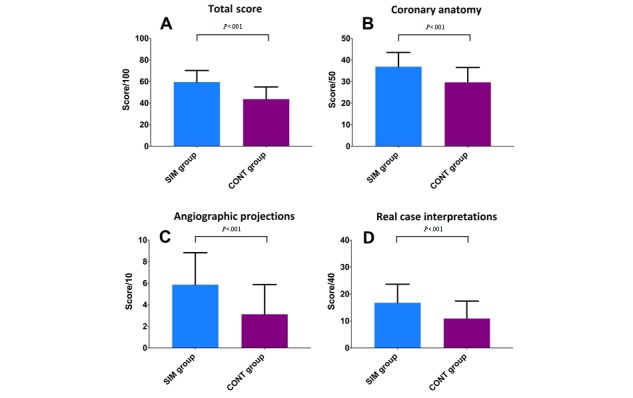
Score according to group allocation at each part of the evaluation (simulant group [SIM] vs control group [CONT]). A) total score; B) coronary anatomy questions (part 1); C) angiographic projections (part 2); and D) real case interpretations (part 3). *P* value determined by Student *t* test.

Overall, the students in the SIM group had higher scores compared with students in the CONT group: 59.8 (SD 11.2) points versus 43.8 (SD 10.9) points, respectively (*P*<.001). Interestingly, students in the SIM group scored higher in each subsection of the evaluation (part 1—coronary anatomy: 36.9 [SD 6.7] points vs 29.7 [SD 7.12] points, *P*<.001; part 2—spatial representation: 6.0 [SD 3.1] points vs 3.0 [SD 2.8] points, *P*<.001; and part 3—interpretation of real angiographies: 16.9 [SD 7.3] points vs 11.2 [SD 6.1] points, *P*<.001; see Figure 3).

Student satisfaction was excellent in both the groups, but higher in the SIM group (98%, 58/59) compared with the CONT group (75%, 44/59; *P*<.001; see Table 2).

**Table 1:** Baseline characteristics of the population.

Variable		All students (N=118)	CONT^a^ group (n=59)	SIM^b^ group (n=59)
Age (in years) at inclusion, mean (SD)		22.6 (1.4)	22.6 (1.3)	22.6 (1.5)
Sex (female), n (%)		62 (52.5)	31 (52.5)	31 (52.5)
**Year of study, n (%)**				
	Fourth	42 (35.6)	21 (35.6)	21 (35.6)
	Fifth	42 (35.6)	21 (35.6)	21 (35.6)
	Sixth	34 (28.8)	17 (28.8)	17 (28.8)

^a^CONT group: control group (traditional teaching).

^b^SIM group: simulator group (simulation teaching).

**Table 2:** Results of the evaluation according to group allocation.

Variable	CONT^a^ group (n=59)	SIM^b^ group (n=59)	*P* value
Part 1 score (out of 50), mean (SD)	29.6 (6.9)	36.9 (6.6)	<.001^c^
Part 2 score (out of 10), mean (SD)	3.1 (2.8)	5.9 (3.0)	<.001^c^
Part 3 score (out of 40), mean (SD)	10.9 (6.5)	16.8 (6.9)	<.001^c^
Total score (out of 100), mean (SD)	43.7 (11.3)	59.5 (10.8)	<.001^c^
Satisfaction, n (%)	44 (75)	58 (98)	<.001^d^

^a^CONT group: control group (traditional teaching).

^b^SIM group: simulator group (simulation teaching).

^c^*P* value determined by Student *t* test.

^d^*P* value determined by chi-square test.

## Discussion

### Principal Findings

This prospective randomized study represents one of the first attempts to assess the effectiveness of a simulator-based approach to teach cardiology to medical students. We report improved scores for students with simulation teaching compared with those attending a traditional course, irrespective of the type of evaluation and the year of medical school. This suggests that coronary anatomy, CA projections (knowledge) are better taught through a simulator-based strategy and that it could translate to a better analysis of real clinical cases (medical skills).

In France, coronary anatomy is taught to medical students in the second year, only before the validation of a Medical Degree. This can explain the absence of any difference between the scores of students according to their years of medical school in our study.

The use of simulators in medical education has vastly increased in recent years [6], and there are now a wide variety of commercially available products, both low-fidelity and high-fidelity. A thorough analysis of simulation studies in all branches of medicine suggests that high-fidelity simulations can facilitate learning, given the appropriate setting, but most studies have no control group [7,8]. One study comparing a simulation approach for the teaching of perioperative ultrasound to anesthesiology residents [9], for example, indicated that it might be a more effective approach than didactic teaching. A previous study in cardiology showed that even a brief experience on a simulator can serve to better prepare the novice cardiology fellow for a range of potential procedures and procedural complications [10]. Overall, however, the level of evidence of these studies is weak, and there are no studies with a robust methodology assessing the interest of simulator teaching in cardiology.

The students in both of our study groups (simulation and traditional teaching) first had a 30-min educational session. They then underwent the same evaluation that was conducted on a website in a blinded manner. Thus, the teaching and evaluation times were identical for both the groups, limiting the evaluation bias.

We believe that the better scores obtained by the students undergoing simulation teaching could be related to the manipulation of the simulator in the SIM group where students could experience a variety of incidences. They could, therefore, acquire a better understanding of the 3D structure, which may facilitate data retention by transfer [11].

One of the recurrent problems in medicine is the transformation of book-based knowledge into practical skills. SIM students are better at analyzing real angiographic films. However, theoretical medical training is not limited to recognizing arteries during an MCQ but aims to integrate its knowledge into practice to improve patient care in daily practice.

Another parameter that could improve teaching is the playful side provided by the simulator.

Indeed, during the session, each medical student in the SIM group was immersed in a virtual reality using realistic tools comparable with a game controller to perform a CA as an active player. These students, with no experience in interventional cardiology, were directly involved in their learning thanks to the simulator and drawn *into the game*, making transmission of medical knowledge and skills easier. Previous studies showed that a playful environment, such as a video game, encourages student’s participation and improves retention and satisfaction rate [12].

Most data about simulation approaches focus on young doctors without experience, notably in surgery or interventional techniques, and show an improvement of skills with simulation training [13]. Our study was different in 2 main parameters. The first was that we assessed a population of medical students rather than medical doctors. Therefore, this study was their first exposure to interventional cardiology. The second was the design of our study, which was not a training study because each student in the SIM group performed 1 single session, more like a serious game than a simulator-based training. Indeed, our teaching study compared 2 different methods with identical time input, whereas in training studies, the duration of exposure to the simulator varies.

Some investigators have demonstrated that high-fidelity simulation may serve as a viable didactic platform for preclinical medical education with improvement in mid- and long-term knowledge retention in comparison with traditional teaching [14].

### Limitations and Future Directions

Our randomized trial has some limitations, including the lack of sample size calculation, the limited size, and the absence of long-term evaluation. Immediate evaluation after a teaching course does not assess medium- and long-term memorization of coronary anatomy and may promote traditional teaching at the expense of simulation teaching. Re-evaluating students after a short period (at least 1 month after the teaching course) could generate some interesting data.

No pretesting was done before the randomization for logistic reasons. However, we performed a randomization to have 2 comparable groups and exclude a selection bias. Moreover, we did not test low-fidelity models of simulation. These less expansive types of simulation had to be compared with traditional teaching and high-fidelity simulation, before considering their generalization.

### Conclusions

In summary, compared with traditional teaching, we found that high-fidelity simulator-based teaching in CA significantly improves students’ knowledge of coronary arteries anatomy, spatial representation, and interpretation of real clinical cases.

Besides improving theoretical knowledge of students in cardiology, simulator-based teaching could improve clinical skills of the students, because our aim is to focus on training students to become caregivers rather than exclusively being founts of knowledge. Despite the high cost of the simulator, simulation teaching in the cardiology student’s program could improve their medical knowledge and potentially medical skills. However, other studies with rigorous methodology should be conducted to evaluate the impact of simulation teaching in various medical specialties.

## Abbreviations

3D: 3-dimensional
CA: coronary angiography
CONT: control teaching
MCQ: multiple choice question
SIM: simulator

## Appendix

Multimedia Appendix 1 CONSORT‐EHEALTH checklist (V 1.6.1).
